# Discriminating between Terminal- and Non-Terminal Respiratory Unit-Type Lung Adenocarcinoma Based on MicroRNA Profiles

**DOI:** 10.1371/journal.pone.0160996

**Published:** 2016-08-30

**Authors:** Mi-Hyun Kim, Jeong Su Cho, Yeongdae Kim, Chang Hun Lee, Min Ki Lee, Dong Hoon Shin

**Affiliations:** 1 Department of Internal Medicine, School of Medicine, Pusan National University, Busan, Republic of Korea; 2 Department of thoracic surgery, School of Medicine, Pusan National University, Busan, Republic of Korea; 3 Department of Pathology, School of Medicine, Pusan National University, Yangsan, Republic of Korea; 4 Medical Research Institute, Pusan National University, Busan, Republic of Korea; University of Connecticut Health Center, UNITED STATES

## Abstract

Lung adenocarcinomas can be classified into terminal respiratory unit (TRU) and non-TRU types. We previously reported that non-TRU-type adenocarcinoma has unique clinical and morphological features as compared to the TRU type. Here we investigated whether micro (mi)RNA expression profiles can be used to distinguish between these two subtypes of lung adenocarcinoma. The expression of 1205 human and 144 human viral miRNAs was analyzed in TRU- and non-TRU-type lung adenocarcinoma samples (n = 4 each) by microarray. Results were validated by quantitative real-time (qRT-)PCR and in situ hybridization. A comparison of miRNA profiles revealed 29 miRNAs that were differentially expressed between TRU- and non-TRU adenocarcinoma types. Specifically, hsa-miR-494 and ebv-miR-BART19 were up regulated by > 5-fold, whereas hsa-miR-551b was down regulated by > 5-fold in the non-TRU relative to the TRU type. The miRNA signature was confirmed by qRT-PCR analysis using an independent set of paired adenocarcinoma (non-TRU-type, n = 21 and TRU-type, n = 12) and normal tissue samples. Non-TRU samples showed increased expression of miR-494 (p = 0.033) and ebv-miR-BART19 (p = 0.001) as compared to TRU-type samples. Both miRNAs were weakly expressed in the TRU type but strongly expressed in the non-TRU type. Neither subtype showed miR-551b expression. TRU- and non-TRU-type adenocarcinomas have distinct miRNA expression profiles, suggesting that tumorigenesis in lung adenocarcinoma occur via different pathways.

## Introduction

Non-small-cell lung cancer (NSCLC) is a lethal disease and the leading cause of cancer-related deaths worldwide [1]. Adenocarcinoma is the most common type of lung cancer, accounting for 40% of all NSCLC cases [2, 3]. The incidence of lung adenocarcinoma is increasing in many countries [4].

Adenocarcinoma can be histologically classified as terminal respiratory unit (TRU) and non-TRU types [5–7]. The former originates from type II pneumocytes or Clara cells and is associated with thyroid transcription factor (TTF)-1 expression [6], with well-defined clinicopathological characteristics [5–7]. Cytologically, cells have protruding or hobnail cytoplasm and frequently harbor a mutation in the epidermal growth factor receptor (*EGFR*) gene. In contrast, non-TRU-type adenocarcinoma was previously only presumed until we described its pathogenesis and clinicopathological features [8, 9]. This subtype arises from ciliated columnar cells that undergo mucous columnar cell changes and subsequent dysplasia; it is also characterized by mucin (MUC)5AC or MUC5B expression, distinguishing it from TRU-type adenocarcinoma [8, 9]. Importantly, the non-TRU type is associated with a poorer prognosis, as confirmed by our group and others [8–10].

The distinction between the two subtypes of adenocarcinoma remains poorly defined at the molecular level. This is due in part to the lack of fresh tissue samples for gene expression analyses owing to the rarity of the non-TRU subtype. In the present study, we compared micro (mi)RNA expression profiles of the two subtypes since miRNAs are relatively well-preserved in formalin-fixed paraffin-embedded tissue. Moreover, recent studies have reported that miRNA expression is useful for examining the molecular characteristics of cancer [11].

## Materials and Methods

### Case selection and histological review

Non-TRU- and TRU-type adenocarcinoma cases (n = 21 and 12, respectively) were included in the analysis. Of the 21 non-TRU cases, eight were selected from our previous study based on the availability and condition of paraffin tissue blocks, and the other 13 cases were newly retrieved from the lung adenocarcinoma case archives at Pusan National University and Pusan National University Yang San Hospital (2010–2014). Slides were reviewed by three pathologists (DHS, CHL, and KUC) based on previously defined criteria. Briefly, the presence of Clara cells, and type II pneumocytes with dome-shaped and protruding cytoplasm was indicative of the TRU type, while adenocarcinomas in which cells exhibited a tall cytoplasm, flat apical border, and continuity with mucous columnar cells were classified as the non-TRU subtype. This study was conducted with approval from the Institutional Review Board of the Pusan National University Hospital (E-2013006).

### MiRNA microarray analysis

TRU- and non-TRU-type adenocarcinoma tissue samples (n = 4 each) obtained by surgical resection from 2005 to 2012 at Pusan National University Hospital were analyzed by microarray. The biospecimens and data used for this study were provided by the Biobank of Pusan National University Hospital, a member of the Korea Biobank Network. Formalin-fixed, paraffin-embedded tissue cores were deparaffinized with xylene at 50°C for 3 min. Total miRNA was extracted from tissues and purified using the mirVana miRNA Isolation kit (Applied Biosystems/Ambion, Austin, TX, USA) according to the manufacturer’s instructions, then labeled and hybridized with the Human microRNA Microarray (Agilent Technologies, Santa Clara, CA, USA)—which includes 1205 human and 144 human viral miRNA genes—according to the manufacturer’s protocol for Agilent microRNA microarray v.2.2. Signals were detected on a DNA microarray scanner (Agilent Technologies), and scanned images were analyzed using Agilent feature extraction software (v.10.10.1.1).

### Quantitative real-time (qRT-)PCR of mature miRNAs

Results obtained by microarray analysis were validated by qRT-PCR. MiRNA expression levels were quantified using the TaqMan MicroRNA Assay (Applied Biosystems) according to the manufacturer’s instructions, with some modifications. Briefly, 20 ng of total RNA were reverse transcribed using the TaqMan MicroRNA RT kit (Applied Biosystems). Reactions contained 10× buffer, 0.15 μl of 100 mM dNTP with dTTP, 0.19 μl RNase inhibitor (20 U/μl), 0.8 μl of MultiScribe reverse transcriptase (50 U/μl), 3 μl miRNA-specific stem-loop primer [002365 (has-miR-494), 001535 (has-miR-551b), and 197235_mat (ebv-miR-BART19)] (Applied Biosystems), and 5 μl input RNA in a total volume of 1.5 μl. Mixtures were incubated at 16°C for 30 min, followed by 42°C for 30 min, and then 85°C for 5 min. qRT-PCR was carried out on a Mx3005P QPCR System (Stratagene, La Jolla, CA, USA) in a 20-μl reaction volume containing 20× TaqMan MicroRNA assay with PCR primers and probes (with 5' 6-carboxyfluorescein and 3' tetramethylrhodamine), 1 μl of the reverse-transcribed product diluted 1:2 in nuclease-free water, and 10 μl of 2× TaqMan Universal Master mix (No AmpErase UNG) (Applied Biosystems). The reaction was incubated at 50°C for 2 min and 90°C for 10 min, followed by 50 cycles of 95°C for 15 s and 60°C for 1 min. Data were analyzed with MxPro–Mx3005P v.3.00 (Stratagene) with the automatic comparative threshold cycle (Ct) setting for adapting baseline values and determining the Ct threshold. Expression levels of mature miRNAs were normalized to that of U6 (2^−ΔCt^), and the fold change in each miRNA was calculated from differences in expression level between tumor and normal tissues.

### In situ hybridization (ISH)

ISH for miRNA detection was carried out using a miRCURY LNA microRNA ISH Optimization kit (Exiqon, Vedbaek, Denmark) and digoxigenin (DIG)-labeled hsa-miR-126 detection probe (Exiqon) at a final concentration of 50 nmol/l. Proteinase K treatment, probe hybridization, and stringent washing were carried out according to the manufacturer’s protocol. Hybridization was performed at 55°C for 1 h, with a scrambled microRNA probe (Exiqon) used as a negative control. The hybridized DIG-labeled probe was detected using sheep anti-DIG-alkaline phosphatase (Roche Applied Science, Basel, Switzerland) and nitro-blue tetrazolium/5-bromo-4-chloro-3'-indolylphosphate ready-to-use tablets (Roche Applied Science). ISH for Epstein–Barr virus (EBV)-encoded mRNA (EBER) was performed in a Bond-Max autostainer (Vision Biosystems, Melbourne, Australia) with an EBV-encoded RNA probe from Leica (Newcastle, UK) according to the manufacturer’s instructions. Scoring of ISH was performed semiquantitatively based on staining intensity: -, absent; +, weak (visible at 200x); ++, moderate (visible at 100x magnification); +++, strong (visible at 40 magnification). Absence or weak expression was regarded as low expression and moderate to strong as high expression.

### Statistical analysis

Given the small sample sizes, miRNA levels in tumor and normal tissues were compared with the Mann–Whitney U test. SPSS v.18.0 for Windows software (SPSS Inc., Chicago, IL, USA) was used for statistical analysis. *P* < 0.05 was considered as statistically significant.

## Results

### Cytological features of TRU- and non-TRU-type lung adenocarcinomas

**Fig 1:**
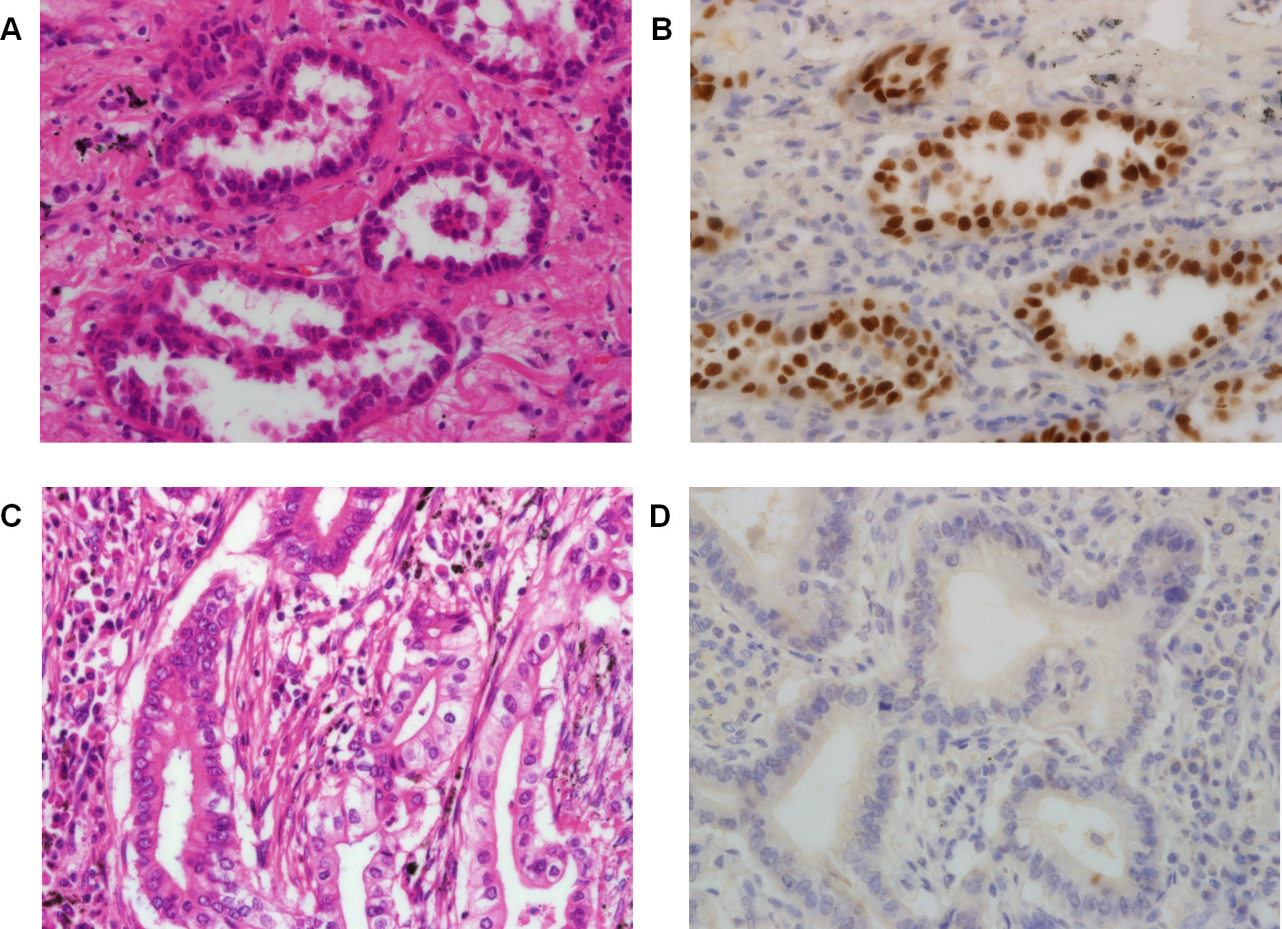
Cytological characteristics of TRU- and non-TRU-type lung adenocarcinoma. (A) TRU-type adenocarcinoma cells have dome-shaped cytoplasm. (B) TRU-type adenocarcinoma cells are strongly positive for TTF-1. (C) Non-TRU-type adenocarcinoma cells have a flat apical cytoplasm. (D) Non-TRU-type adenocarcinoma cells are negative for TTF-1.

Tumor cells in TRU-type adenocarcinoma had a dome-shaped or protruding cytoplasm (Fig 1A) and showed strong but diffuse expression of TTF-1 (Fig 1B). In contrast, tumor cells in non-TRU-type adenocarcinoma had cuboidal or columnar cytoplasm with a flat apical border (Fig 1C) and were TTF-1-negative (Fig 1D).

### TRU- and non-TRU-type lung adenocarcinoma miRNA expression profiles

**Fig 2:**
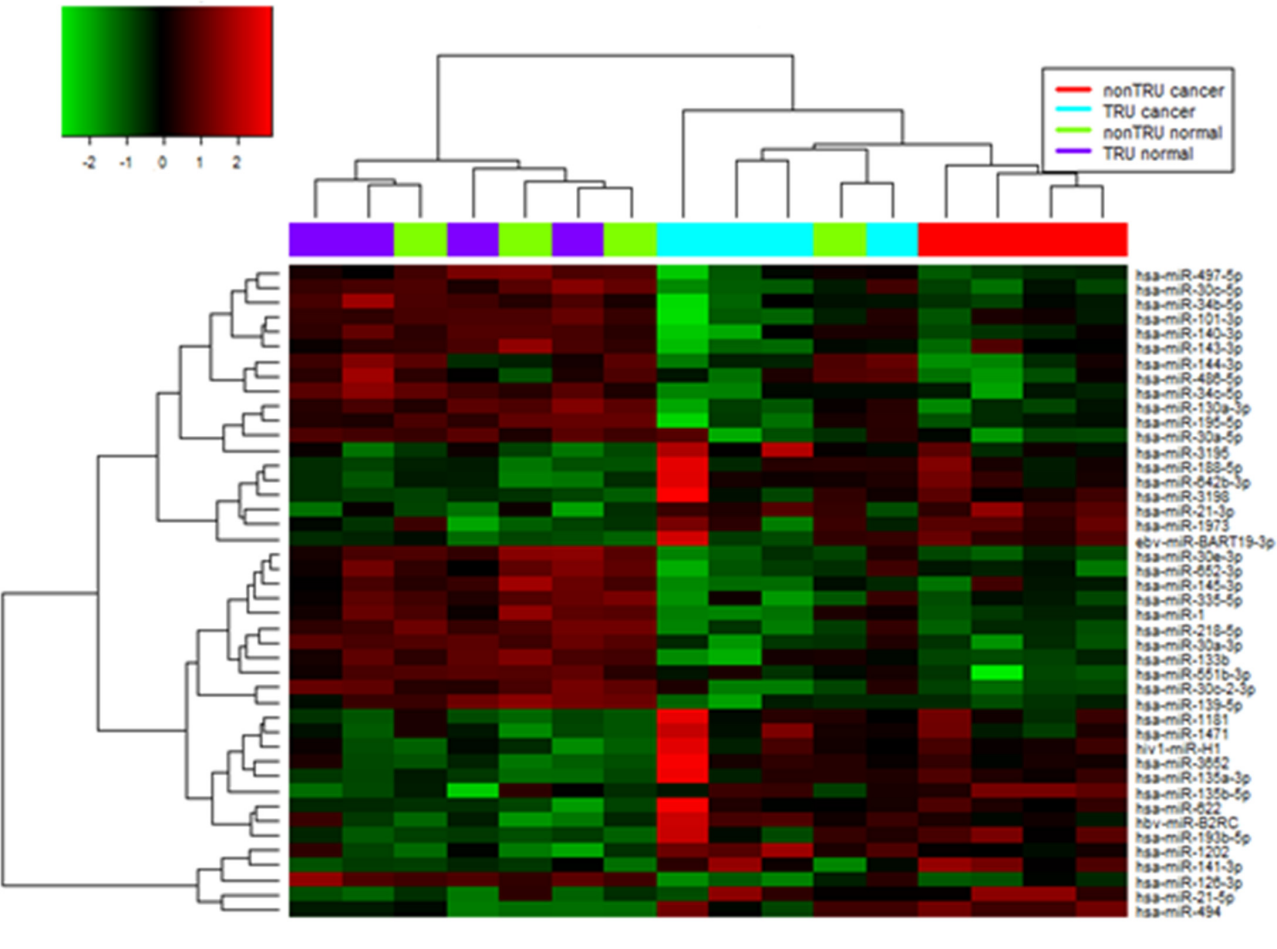
MiRNAs differentially expressed between lung adenocarcinoma vs. normal lung tissue. Hierarchical clustering of 44 miRNA genes with significantly different expression (p<0.05) in tumor tissues. Rows and columns represent individual genes and tissue samples, respectively. The scale represents the intensity of gene expression (log2 ranges between −2.0 and 2.0).

MiRNA profiles of eight pairs of primary lung cancer and corresponding normal lung tissue samples were determined using an array of 1205 human and 144 human viral miRNAs. A total of 44 miRNAs showed significant differences in expression between tumor and normal tissues (Fig 2). One miRNA was upregulated and 12 were down regulated in TRU-type samples relative to corresponding normal lung tissue (Table 1). For the non-TRU subtype, 11 miRNAs were upregulated and nine were down regulated in tumor as compared to normal tissues (Table 2). The expression of 29 miRNAs differed significantly between TRU- and non-TRU-type samples. In particular, hsa-miR-494 and ebv-miR-BART19 were upregulated by > 5-fold (8.03- and 6.98-fold, respectively), whereas hsa-miR-551b was down regulated by 7.30-fold in non-TRU as compared to TRU-type adenocarcinoma. Only two miRNAs (hsa-miR-1 and hsa-miR-133b) were down regulated in both adenocarcinoma subtypes relative to normal tissue.

**Table 1:** Thirteen differentially expressed microRNAs in terminal respiratory unit type lung cancer tissues and normal lung tissues.

microRNA	Location	Type (fold change)	p Value
hsa-miR-1	18q11.2	Down (-5.95)	0.012
hsa-miR-1202	6p22.3	Up (3.76)	0.048
hsa-miR-126	9q33.3	Down (-4.57)	0.034
hsa-miR-133b	6p12.3	Down (-5.30)	0.025
hsa-miR-139	11q13.4	Down (-4.30)	0.008
hsa-miR-140	16q22.1	Down (-2.51)	0.047
hsa-miR-143	5q32	Down (-2.95)	0.040
hsa-miR-145	5q32	Down (-3.01)	0.015
hsa-miR-30a	6q13	Down (-4.05)	0.040
hsa-miR-30c	6q13	Down (-3.18)	0.040
hsa-miR-34b	11q23.1	Down (-6.37)	0.032
hsa-miR-34c	11q23.1	Down (-8.23)	0.018
hsa-miR-4324	19q13.42	Down (-2.08)	0.025

**Table 2:** Twenty differentially expressed microRNAs in non-terminal respiratory unit type lung cancer tissues and normal lung tissues.

microRNA	Location	Type (fold change)	p Value
ebv-miR-BART19		Up (6.98)	0.027
hbv-miR-H1		Up (2.14)	0.042
hsa-miR-1	18q11.2	Down (-5.20)	0.012
hsa-miR-1246	2q31.1	Up (2.57)	0.049
hsa-miR-1290	1p36.13	Up (3.01)	0.036
hsa-miR-133b	6q12.3	Down (-4.29)	0.019
hsa-miR-135a-3p	3p24.3	Up (3.27)	0.033
hsa-miR-135b	1q32.1	Up (3.24)	0.025
hsa-miR-141	12p12.3	Up (2.52)	0.021
hsa-miR-195	17p13.1	Down (-2.60)	0.023
hsa-miR-200b	1p36.33	Up (2.72)	<0.001
hsa-miR-210	11p15.5	Up (3.23)	0.021
hsa-miR-30b	8q24.2	Down (-2.02)	0.015
hsa-miR-30c	1p34.2	Down (-2.32)	0.039
hsa-miR-494	14q32.3	Up (8.03)	0.021
hsa-miR-497	17p13.1	Down (-2.15)	0.014
hsa-miR-502	Xp11.23	Down (-2.43)	0.014
hsa-miR-532	Xp11.23	Down (-2.11)	0.049
hsa-miR-551b	3q26.2	Down (-7.30)	0.034
hsa-miR-622	13q31.1	Up (2.39)	0.015

### Validation of microarray data by qRT-PCR analysis

**Fig 3:**
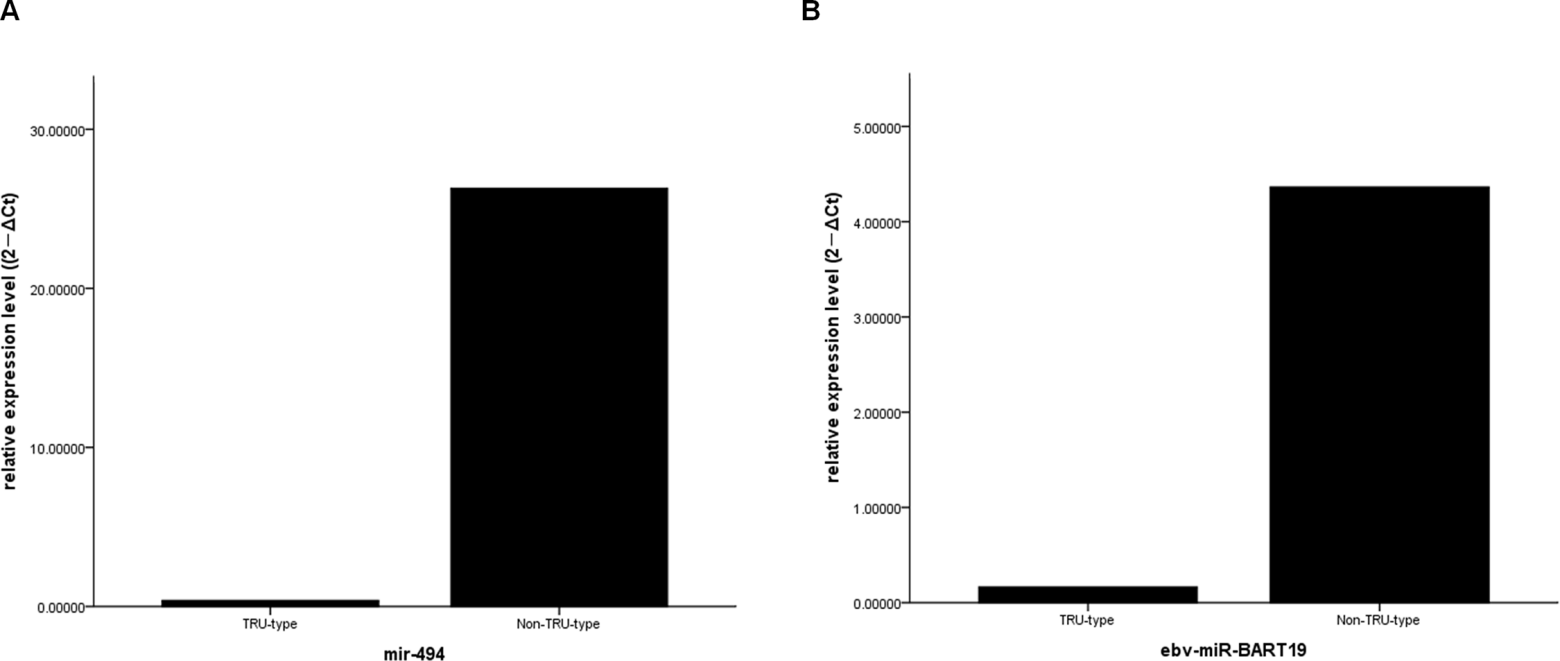
Validation of the microarray data by real-time quantitative RT-PCR. (A) The levels of the mature forms of miR-494 was significantly increased in non-TRU-type compared with TRU-type samples (p = 0.033). (B) Ebv-miR-BART19 were significantly increased in non-TRU-type compared with TRU-type samples (p = 0.001).

Three miRNAs (hsa-miR-494, hsa-miR-551b, and ebv-miR-BART19) were validated in an independent sample set of non-TRU- and TRU-type lung adenocarcinoma and corresponding normal lung tissue (n = 21 and 12, respectively) by qRT-PCR. The levels of mature has-miR-494 was higher in non-TRU- relative to TRU-type samples (p = 0.033, Fig 3A). Also, ebv-miR-BART19 was higher in non-TRU- relative to TRU-type samples (p = 0.001, Fig 3B).

### Lung adenocarcinoma patient characteristics

The baseline characteristics of the 33 patients enrolled in this study are summarized in Table 3. The mean age of patients was 65 years and 18 patients (54.5%) were male. There were more smokers in the non-TRU-type than in the TRU-type adenocarcinoma group. *KRAS* and *EGFR* mutations were detected in one (8.3%) and four (33.3%) TRU-type patients, respectively, as compared to six (30.0%) and two (10.0%) patients, respectively, in the non-TRU-type group.

**Table 3:** Patient characteristics.

Variables	TRU (n = 12)	Non-TRU (n = 21)	p Value
Age (mean±SD)	65±10	64±8	0.676
Sex, male (%)	7 (58)	11 (52)	0.514
T stage (%)			0.006
1	10 (83)	5 (24)	
2	2 (17)	5 (24)	
3	0	10 (48)	
4	0	1 (4)	
N stage			0.126
0	9 (75)	14 (67)	
1	3 (25)	2 (9)	
2	0 (0)	5 (24)	
Smoking (%)			0.183
Never	9 (75)	11 (52)	
Ever	3 (25)	10 (48)	
Smoking, PY (mean±SD)	6±13	25±32	0.027
*EGFR* (%)	4 (33)	2 (10)	0.122
*KRAS* (%)	1 (8)	6 (30)	0.161

TRU, terminal respiratory; PY, pack year.

### Evaluation of miRNA expression by ISH

**Fig 4:**
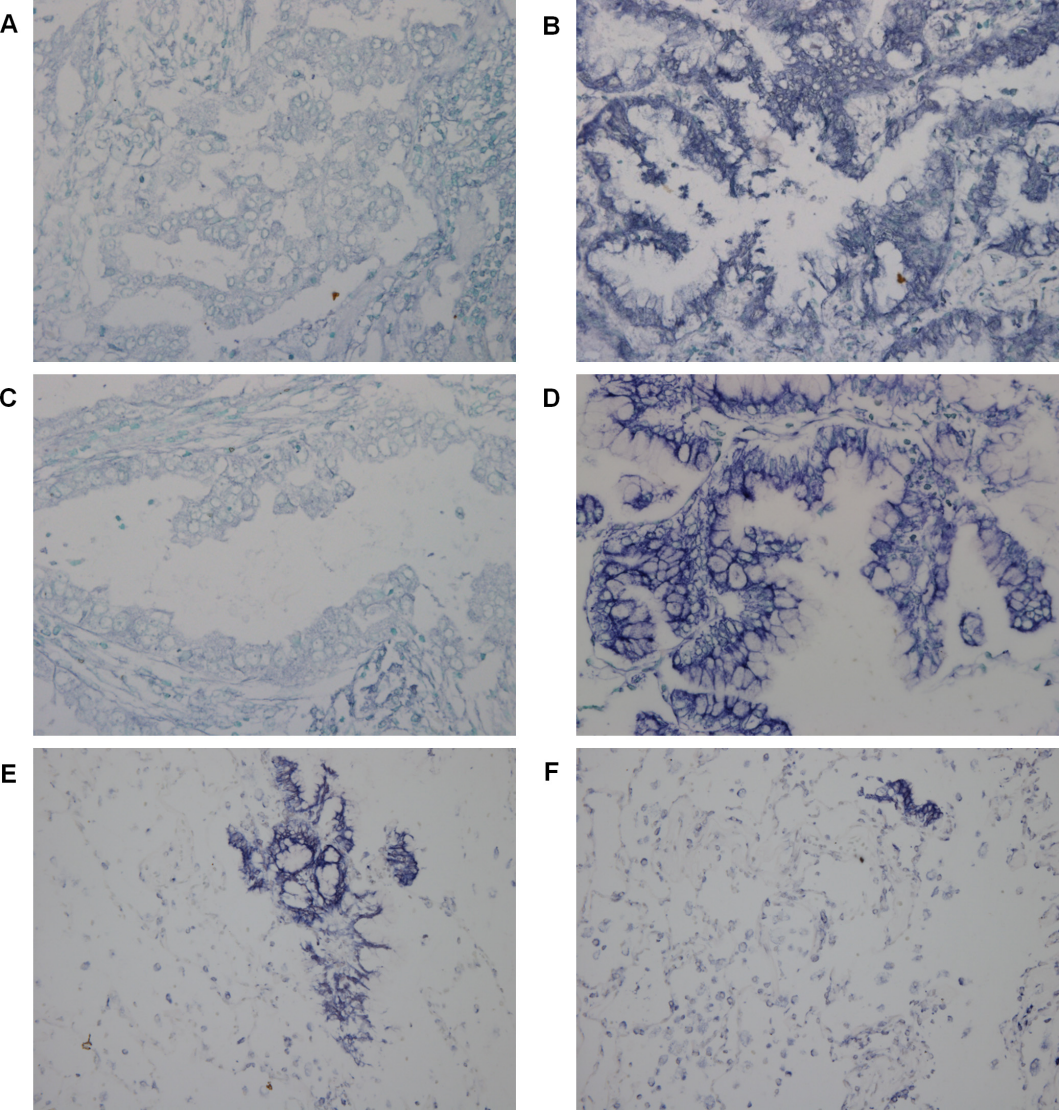
Detection of miRNA expressions by ISH. (A) MiR-494 was weakly expressed in TRU-type. (B) MiR-494 was strongly expressed in non-TRU-type adenocarcinoma. (C) Ebv-miR-BART19 was weakly expressed in TRU-type. (D) Ebv-miR-BART19 was strongly expressed in non-TRU-type adenocarcinoma. (E) MiR-494 was weakly expressed in normal lung tissue. (F) Ebv-miR-BART19 was weakly expressed in normal lung tissue.

MiR-494 expression was low in TRU- but high in non-TRU-type adenocarcinoma (Fig 4A and 4B). A similar trend was observed for ebv-miR-BART19 (Fig 4C and 4D). Both of miR-494 (Fig 4E) and ebv-miR-BART19 (Fig 4F) were only weakly expressed in normal lung tissue. MiR-551b was not detected in either subtype. MiR-494 was expressed in the bronchial epithelium in the non-TRU subtype. Results obtained by ISH revealed that all cases were negative for EBV.

## Discussion

Lung adenocarcinoma is a heterogeneous group of diseases diagnosed and classified into subtypes according to the World Health Organization classification. TTF-1-positive adenocarcinomas exhibit a high degree of cytological similarity to, and may arise from, type II pneumocytes or Clara cells [7]. This type of lesion is known as TRU-type adenocarcinoma and is prevalent among females and non-smokers, and is associated with *EGFR* mutation [5]. In contrast, non-TRU-type adenocarcinoma has not been well characterized. We previously reported that this subtype arises from ciliated columnar cells via mucous columnar cell metaplasia [8] and exhibits mucinous features, with positive expression of MUC5AC and MUC5B [9–10].

Our previous studies focused mainly on the pathological and clinical aspects of the disease. In this study, we confirmed non-TRU-type adenocarcinoma as being distinct from the TRU type based on molecular features—namely, their miRNA expression profiles, which revealed 29 miRNAs that are differentially expressed between the subtypes. Only two miRNAs (hsa-miR-1 and hsa-miR-133b) were shared by both TRU- and non-TRU-type adenocarcinoma; two others—hsa-miR-494 and ebv-miR-BART19—were upregulated by > 5-fold whereas hsa-miR-551b was downregulated by > 5-fold in the non-TRU type relative to the TRU type, confirming that they are histologically and molecularly dissimilar. Recognizing this distinction is important for several reasons. Firstly, the non-TRU type has worse prognosis than the TRU type; this is a predictable consequence of the higher frequency of *KRAS* mutation in the former [8–10]. Secondly, lung adenocarcinoma is highly heterogeneous, and classifying it into homogeneous subgroups will enable the development of targeted therapies.

MiR-494 is an oncomiR in gastrointestinal stromal tumors that targets the *KIT* proto-oncogene [12]. It also enhances myocyte survival by targeting *phosphatase and tensin homolog* (*PTEN*), *Rho-associated protein kinase 1*, and *calmodulin kinase IId*, against ischemia/reperfusion-induced cardiac injury [13]. Mir-494 has also been shown to regulate the accumulation and activity of myeloid-derived suppressor cells by targeting PTEN-mediated activation of the AKT signaling pathway [14]. In NSCLC cell lines, miR-494 has been shown to modulate BIM expression via the ERK1/2 [15]. One study found that miR-494 levels were upregulated in mouse bronchial epithelial cells exposed to benzo[a]pyrene, a well-known carcinogen present in coal tar, cigarette smoke, and smoked foods [16]. Non-TRU-type adenocarcinoma may arise via changes in mucous columnar cells, which can be induced by inflammation caused by environmental pollutants or smoking. Indeed, in the present study, a higher percentage of patients with this adenocarcinoma subtype were smokers as compared to TRU-type patients.

The microarray, qRT-PCR, and ISH analyses identified ebv-miR-BART19 as being upregulated in non-TRU-type adenocarcinoma. However, we failed to detect the presence of EBV in tumor cells or lymphocytes around the tumor in both adenocarcinoma subtypes. There are a few articles, investigating viral miRNAs in cancer [17, 18]. They showed EBV miRNAs can be upregulated in solid cancers with the absence of EBV-encoded small RNAs (EBER). There are a few possible reasons why EBV miRNAs were found in the absence of EBERs. Firstly, it may have arisen from cross-reaction with other miRNAs; secondly, the assays may be so sensitive that even rare infected cells give positive results; and finally, EBV miRNAs from infected lymphocytes may be delivered to tumor cells. Whatever the most accurate model is, upregulation of ebv-miR-BART-19 in non-TRU-type adenocarcinoma may imply the different pathogenesis of non-TRU-type adenocarcinoma and TRU-type adenocarcinoma such as an association with inflammation. As we previously reported, non-TRU-type adenocarcinoma have poorer prognosis than TRU-type adenocarcinoma [8, 9]. This finding may be partially explained by the study showing patients with viral miRNAs have relatively poor survival [18]. They showed that these patients were featured by increased expression of programmed death 1 (PD-1)/ programmed death-ligand 1 (PD-L1), a pathway implicated in tumors escaping immune destruction. It is consistent with prior study, reported that herpetic viral reactivation is associated with stimulation of the PD-1/PD-L1 pathway [19]. Also prior reports of EBV-infected cells showed the increased expression of cytokines known to inhibit host response to cancer [18, 20–22].

The downregulation of miR-551b in non-TRU-type as compared to TRU-type adenocarcinoma observed by microarray analysis was not confirmed by qRT-PCR or ISH, suggesting that it is expressed at very low levels. Alternatively, the microarray analysis may have yielded falsely positive results.

In addition to the three miRNAs that were differentially expressed between TRU-type and non-TRU-type adenocarcinomas, we found two miRNAs—miR-1 and miR-133b—that were downregulated in both subtypes. MiR-1 has been reported as a tumor suppressor in various cancers including NSCLC. One study showed that miR-1 inhibits the tumorigenic properties of lung cancer cells by targeting Slug, a transcriptional repressor of E-cadherin and an inducer of epithelial-to-mesenchymal transition [23]. MiR-1 was also suggested to play an important role in the pathogenesis of NSCLC by regulating PIK3CA catalytic subunit alpha via the PI3K/Akt pathway [24]. MiR-133b expression was found to be reduced in lung cancer tissue [25, 26], and was shown to regulate cell growth, invasion, and apoptosis via regulation of EGFR expression [26]. Although our findings are consistent with those of previous studies, additional research is needed in order to clarify the precise roles of these miRNAs in lung cancer.

In conclusion, we demonstrated that TRU- and non-TRU-type adenocarcinomas have distinct miRNA expression profiles, suggesting that they likely induce tumorigenesis via different mechanisms. We also identified miR-494 as a potential molecular marker for non-TRU-type adenocarcinoma. The number of cohort in the present study is not big, hence our findings are still preliminary and further studies are definitely necessary to confirm by ourselves and other researchers repeatedly. Our future studies will focus on identifying molecular profiles with open source data such as The Cancer Genome Atlas (TCGA) to verify our findings on larger cohort. Simultaneously, it needs to find the target genes of these miRNAs to better understand the origin of non-TRU-type adenocarcinoma.
